# An analysis of the intestinal microbiome combined with metabolomics to explore the mechanism of how Pangxiejiao tea improves metabolic disorders in HFFD-treated rats

**DOI:** 10.3389/fnut.2025.1653855

**Published:** 2025-09-17

**Authors:** Wanchun Wang, Enzheng Zhu, Yang Yang, Qingqing Huang, Xue Xiao, Shenghua Piao

**Affiliations:** ^1^Guangdong Metabolic Diseases Research Center of Integrated Chinese and Western Medicine (Institute of Chinese Medicine), Guangdong Pharmaceutical University, Guangzhou, China; ^2^Guangdong Provincial Key Laboratory of Chinese Medicine for Metabolic Diseases, Guangdong Pharmaceutical University, Guangzhou, China

**Keywords:** Pangxiejiao, high-fat and high-fructose diet, metabolic disorders, gut microbiota, metabolomics

## Abstract

**Introduction:**

The high-fat and high-fructose diet (HFFD) can lead to various metabolic disorders. Pangxiejiao (PXJ), a natural plant widely used in folk practices, has been found to contain bioactive compounds that exhibit hypoglycemic effects *in vitro*. However, the potential of PXJ aqueous extract to ameliorate metabolic disorders *in vivo* and its underlying mechanisms remain unexplored. This study aims to investigate the effects of PXJ on metabolic disorders induced by HFFD in rats.

**Methods:**

An HFFD was employed to mimic unhealthy dietary habits, while PXJ was administered via oral gavage for 10 weeks. Perform biochemical assays, observe histopathological structures, and evaluate metabolic status in rats to investigate intrinsic alterations through detection of gut microbiota and plasma metabolites.

**Results:**

The results demonstrated that PXJ intervention reduced body weight, improved glucose and lipid metabolism, and decreased serum uric acid levels. PXJ alleviated oxidative stress and inflammation, as evidenced by reduced serum levels of IL-1β, IL-6, and TNF-*α*, along with ameliorated pathological inflammatory manifestations in metabolism-related organs, such as liver, pancreas, and colon. Furthermore, PXJ was found to decrease the *Firmicutes*/*Bacteroidota* ratio, modulated gut microbiota composition, and maintain microbial homeostasis. Nontargeted metabolomics analysis identified 39 upregulated metabolites, including hippuric acid, and 9 downregulated metabolites, such as LysoPG(18:2(9Z,12Z)/0:0). Correlation analysis further revealed that PXJ maintains metabolic homeostasis through complex network crosstalk. Specifically, four gut microbial taxa including Alloprevotella and six metabolites, including hippuric acid, demonstrated significant negative correlations with IL-6, TNF-*α*, and MDA. In contrast, *Lachnospiraceae_NK4A136_group* showed positive correlations with these metabolites and SOD.

**Discussion:**

In conclusion, early and sustained PXJ intervention alleviates HFFD-induced metabolic disorders, which is associated with restored gut microbiota balance, improved metabolism, and reduced inflammation and oxidative stress, demonstrating its potential as a novel functional tea for metabolic disorder prevention.

## 1 Introduction

The high-fat and high-fructose diet (HFFD) represents one of the predominant unhealthy dietary patterns in modern society, closely associated with various metabolic diseases such as diabetes, hyperuricemia, and hyperlipidemia (1–3). Earlier studies predominantly focused on isolated metabolic dysfunctions. A growing body of evidence suggests that HFFD disrupts systemic homeostasis, manifesting as elevated inflammatory markers, gut microbiota dysbiosis, and metabolite imbalance, ultimately leading to the co-occurrence of multiple metabolic disorders (4–6). The mechanisms by which HFFD induces systemic metabolic dysregulation are complex, involving crosstalk between metabolic pathways and multiple organ systems (7, 8). Extensive research has been ongoing to seek more potential solutions.

The consumption of beverages derived from natural plants offers a promising and easily accessible approach to improving bodily metabolism in daily life (9). As a parasitic plant that thrives on various hosts, mistletoe exhibits a range of therapeutic benefits (10), including antihypertensive and hypoglycemic effects, anti-tumor activity, and liver protection (11). Among these, Pangxiejiao (PXJ) stands out as a species of mistletoe (*Viscum articulatum* Burm. f.), which is commonly hosted in ancient tea trees of Yunnan province, southwest China (12). It is also distributed widely in India and Bangladesh (13). Folk practices attribute PXJ with benefits like uric acid reduction, antihypertensive, and liver protection (12, 14), driving its local popularity. PXJ contains bioactive compounds including flavonoids, polysaccharides, triterpenes, alkaloids, among others (14), which may exert anti-inflammatory and antioxidant effects, potentially contributing to the improvement of metabolic disorders. Recently, Zhang et al. found that PXJ extract can inhibit *α*-amylase and α-glucosidase, potentially indicating hypoglycemic activity (15). However, our understanding of whether PXJ can ameliorate systemic metabolic disorders and maintain organismal metabolic homeostasis remains limited.

We employed an HFFD-fed rat model to simulate modern unhealthy dietary patterns. Meanwhile, PXJ was administered as an intervention to investigate its effects on metabolic disorders. To evaluate the effects of PXJ intervention on HFFD-induced metabolic disorders, we systematically measured blood glucose, serum lipid, and serum uric acid levels while simultaneously examining associated pathological changes in visceral tissues. HFFD-induced metabolic disorders involve both gut microbiota dysbiosis and systemic metabolic alterations. Therefore, we assessed inflammatory factors, oxidative stress markers, gut microbiota, and plasma metabolites to analyze their interrelationships and explored the potential mechanisms through which PXJ improves metabolism.

## 2 Materials and methods

### 2.1 Preparation of PXJ water extract

Pangxiejiao (*Viscum articulatum* Burm. f.), is a mistletoe species parasitic on ancient tea trees in Yunnan, China, was purchased from Chenhua Tea Co., Ltd. (Menghai, Xishuangbanna, Yunnan, China). All the dried PXJ were smashed into powder form using a pulverizer and subjected to aqueous extraction. Dried PXJ (1,200 g) was extracted with 12 L of distilled water (1:10, w/v) for 2 h at 100 °C. Following the initial extraction, the same batch of PXJ previously used was subjected to a second extraction under identical conditions using 9.6 L of distilled water (1:8, w/v). Finally, the combined filtrates from both extractions were concentrated under reduced pressure at 60 °C to a final volume of 2.4 L, which was stored at −20 °C for future use.

### 2.2 Determination of intragastric infusion dose

The selected doses align with traditional human consumption levels of 5–10 g/day (16). According to Jian et al., the test substance PXJ medium lethal dose (LD_50_) > 21.5 g/kg, it was a non-toxic substance (17). The gavage dose of rats was calculated according to the “Table of the Dose Conversion Coefficients per Kilogram Body Weight of Animals and Humans.” The conversion coefficient between the adults and rats was 6.3. So, according to the formula of dose = daily normal dose/human weight * 6.3. Thus, the final doses for this study were set at 0.5 g/kg/d (low dose) and 1 g/kg/d (high dose). These doses were used for subsequent experiments.

### 2.3 Animals and groups

The 32 healthy male Sprague–Dawley (SD) rats, aged 6 weeks and weighing between 180 and 200 g, were provided by Spaf-Bio (Beijing, China), with Animal Certificate No. 110324241103856223 and License No. SCXK (Beijing, China) 2024–0001. All animals were housed under standard conditions in the Guangdong Pharmaceutical University Animal Center (SPF environment, temperature 25 ± 2°C, relative humidity 55 ± 10%) and maintained on a 12 h light–dark cycle. Rats were group-housed with four rats per cage. The Animal Ethics Committee of Guang-dong Pharmaceutical University approved the animal care and experimental protocols under the ethical approval number gdpulacspf2022578.

After 1 week of feeding with basal diet and distilled water, the rats were stratified and randomly divided into 4 groups (*n* = 8 per group) based on their average body weight.

Normal control group (CON, basal feed diet and distilled water), HFFD model group (MOD, high-fat diet and fructose solution), PXJ low-dose treatment group (LP, high-fat diet and fructose solution & PXJ 0.5 g/kg/d), PXJ high-dose treatment group (HP, high-fat diet and fructose solution & PXJ 1 g/kg/d). All interventions were delivered daily via oral gavage, with CON group and MOD group animals receiving distilled water. Modeling and PXJ administration were performed simultaneously for a total duration of 10 weeks.

### 2.4 Animal diets and experimental feeding protocols

All rats were allowed free access to food and water. Basal feed diet was obtained from the Guangdong Pharmaceutical University Animal Center. The caloric distribution of the basal diet was as follows: protein 23.07%, fat 11.85%, and carbohydrates 65.08%, with a total energy content of 3.40 kcal/g. The high-fat feed (XTHF45) was obtained from Keda Hengxin Technology Co., Ltd. (Guangzhou, Guangdong, China). The caloric distribution of the high-fat diet was as follows: protein 20.1%, fat 45.1%, and carbohydrates 34.7%, with a total energy content of 4.7 kcal/g. The specific composition of the rat feed is detailed in Supplementary Table S1. The fructose-glucose syrup (F55, with 55% fructose and 45% glucose) was sourced from Shuangqiao Co., Ltd. (Guangzhou, Guangdong, China). The 12% fructose concentration in the HFFD model was chosen to mimic typical fructose intake in modern diets, as supported by prior studies linking this level to metabolic dysregulation (18).

### 2.5 Measurement of food intake, water intake, and body weight

Food and water intake was monitored daily at fixed times by weighing the pro-vided and remaining amounts. The actual food and water intake per rat was calculated using the same formula for both: Actual intake per rat = (Initial amount - Remaining amount)/Number of rats per cage. Supplies were refreshed daily to maintain hygiene. All rats’ body weight was measured on the experiment’s final day.

### 2.6 Oral glucose tolerance test (OGTT)

After 10 weeks of feeding, all rats underwent a 12-h fast and were allowed to drink water. A 50% glucose solution (2 g/kg) was administered orally to all rats. Blood glucose levels were determined at 0, 15, 30, 60, and 120 min using a glucose meter and test strips. The blood glucose measurements calculated the area under the glucose tolerance curve (AUC).

### 2.7 Sample collection

At week 10, all rats were fasted overnight and anesthetized with 2% sodium pentobarbital (0.3 mL/100 g body weight). Blood was collected from the abdominal aorta into heparinized and plain vacuum tubes (5 mL each). After 30 min at room temperature, samples were centrifuged (3,000 rpm, 10 min), and serum/plasma supernatants were aliquoted for analysis. Organs (liver, kidney, pancreas, colon, and adipose tissue) were immediately harvested. Adipose tissue was fixed in a specialized fixative, while other organs were preserved in general fixative (both from Wuhan Pinofei Biological Technology Co., Ltd., China) and stored at 4°C overnight. Colon contents were rapidly aliquoted into enzymefree cryotubes, flash-frozen in liquid nitrogen, and stored at −80°C for further analysis. The samples for gut microbiota analysis and metabolomics testing were collected only once at the endpoint of the intervention.

### 2.8 Biochemical analyses

Serum biochemical parameters were analyzed using commercial kits: FBG, TC, TG, HDL-C, LDL-C, SUA, ALT, AST, BUN, and Scr (Nanjing Jiancheng Bioengineering Institute, China); GHb, FINS, FFA, IL-1β, IL-6, TNF-*α*, MDA, and SOD were quantified using ELISA kits (Jiangsu Meimian Industrial Co., Ltd., China). The assays were performed in strict adherence to the kit manufacturers’ instructions. HOMA-IR and ISI were subsequently calculated.

HOMA-IR = fasting blood glucose (mmol/L) × fasting insulin (mIU/L)/22.5 (19).

ISI = ln [1 / (fasting blood glucose (mmol/L) × fasting insulin (mIU/L))] (20).

### 2.9 Histopathological observation of organs

Visceral fat was collected, blotted dry with sterile filter paper, and weighed to obtain wet mass. Body fat percentage was calculated as: Visceral fat mass (g)/Body weight (g) × 100%.

Tissues (liver, kidney, pancreas, colon, visceral and subcutaneous fat) were fixed in 4% paraformaldehyde at 4°C for 48 h, then paraffin-embedded and sectioned (4 μm) for H&E staining. Liver samples were cryosectioned after liquid nitrogen snap-freezing and stained with Oil Red O (60% isopropanol differentiation, hematoxylin counterstain). All sections were imaged using an Olympus digital microscope. Three random fields were captured for each section at a magnification of 200× and 400×. Regional analysis was conducted using ImageJ software (National Institutes of Health, Bethesda, MD, USA) and measured cell area and the number of cells.

### 2.10 Gut microbiota analysis

#### 2.10.1 DNA detection and PCR product acquisition

Genomic DNA was retrieved from fecal samples using cetyltrimethylammonium bromide (CTAB) and assessed for purity and concentration via 1% agarose gel electrophoresis. The DNA was diluted until the final concentration reached one ng/μL. PCR amplification was performed using primers for the 16 V 34 region (341F,806R; 341F:5’-CCTAYGGGRBGCASCAG-3’, 806R:5’-GGACTACNNGGGTATCTAAT-3’). The PCR reaction mixture included Phusion High-Fidelity PCR Master Mix, 0.2 μM primers, and a 10 ng DNA template. The amplified products were purified using magnetic beads, mixed in equal volumes, and subsequently analyzed.

#### 2.10.2 16S rRNA sequencing and analysis

Libraries were created by applying the NEB Next® Ultra DNA Library Prep Kit and assessed for quality on the Agilent 5,400 platform. Sequencing was carried out on the NovaSeq 6,000 system (Illumina, San Diego, CA, USA). Data were processed by merging paired-end reads, trimming barcode and primer sequences with FLASH (Version 1.2.11) (21), and filtering with fast to obtain high-quality Clean Tags (22). Chimeric sequences were removed by comparing with the SILVA database (version 138.1)^1^ for 16S/18S rRNA genes and the UNITE database for ITS sequences (23), yielding the final Effective Tags. These were processed using QIIME2 software with DADA2 for noise reduction to generate Amplicon Sequence Variants (ASVs). Species annotation was performed, and *α*-diversity indices (observed OTUs, Shannon, Simpson, Chao1) were calculated. Principal Coordinates Analysis (PCoA) and LEfSe analysis were conducted to assess community differences. The technical procedures including DNA extraction, PCR amplification, clone library construction and sequencing were performed by Novogene Technology Co., Ltd. (Beijing, China).

### 2.11 Metabolomics analysis

#### 2.11.1 Metabolite extraction and LC–MS analysis

Plasma samples (100 μL) were combined with ice-cold 80% methanol (v/v) (CAS 67–56-1, Thermo Fisher Scientific, USA) and vortexed for 1 min, followed by centrifugation at 15,000g for 20 min at 4 °C in a D3024R low-temperature centrifuge (D3024R, Scilogex, USA), the supernatant was diluted to 53% methanol and re-centrifuged to remove impurities. Quality control (QC) samples were produced by mixing equal volumes from each group. LC–MS analysis was performed on a Vanquish UHPLC system (Thermo Fisher Scientific, Germany) coupled to a Q Exactive™ HF hybrid quadrupole-Orbitrap mass spectrometer (Thermo Fisher Scientific, Germany). Metabolites were separated on a Hypersil Gold C18 column (100 × 2.1 mm, 1.9 μm, Thermo Fisher Scientific, USA) using a gradient elution of 0.1% formic acid (CAS 64–18-6, Thermo Fisher Scientific, USA) in water (CAS 7732-18-5, Merck, Germany) and methanol at 0.2 mL/min. ESI-MS detection was carried out in positive and negative modes (m/z 100–1,500) (24). All LC–MS analyses were performed by Novogene Technology Co., Ltd. (Beijing, China).

#### 2.11.2 Data processing and differential analysis

Raw data files were translated into mzXML format using ProteoWizard and manipulated with XCMS for peak detection, alignment, and quantification. Metabolite identification was achieved by matching m/z values, retention times, and MS/MS spectra with high-quality spectral databases, using a mass tolerance of 10 ppm. Background noise was removed by analyzing blank samples, and data normalization was performed using the following formula: (sample peak area/total peak area) × (QC1 peak area / total QC1 peak area). Metabolites with a coefficient of variation (CV) greater than 30% in QC samples were excluded to ensure data reliability. Differential metabolites were identified based on VIP > 1, *p* < 0.05, and a fold change greater than 2. Unsupervised Principal Component Analysis (PCA) was carried out to confirm the overall metabolic differences between groups. A supervised Orthogonal Partial Least Squares Discriminant Analysis (PLS-DA) model was developed to improve the resolution of the analysis. The model’s ability to explain and predict was evaluated based on *R*^2^ and *Q*^2^ values. The HMDB database was utilized to define metabolic objects. Potential biomarkers were imported into MetaboAnalyst 6.0^2^ to enrich metabolic differences further and predict metabolic pathways. The HMDB database retrieves related proteins for different metabolites and links them to UniProt to obtain the corresponding gene names. Gene information was searched using UniProt ID in “Genecards” to identify disease-related genes. The “Different Metabolic Related Genes and Metabolic Disorders Related Targets” were fitted in the Venny2.1.0 online tool to find the intersection target. Finally, the intersection target was uploaded to the MetASCAPE database for KEGG pathway enrichment analysis.

### 2.12 Statistical analysis

The statistical analysis was performed using SPSS version 22 (SPSS, Chicago, IL, USA). Normality tests were conducted on the data to verify whether they followed a normal distribution. Continuous variables was analyzed using one-way analysis of variance (ANOVA). The results were analyzed as mean ± standard error of the mean (Mean ± SD) unless stated otherwise. The least significant difference (LSD) *post hoc* test was performed to identify which two groups showed significant differences. Spearman correlation analysis was carried out using the Wekemo Bioincloud platform.^3^ The statistical significance levels were set at *p* < 0.05. GraphPad prism software 10.1.2 (GraphPad Software, San Diego, CA, USA) was used for mapping.

## 3 Results

### 3.1 PXJ improves metabolic disorders induced by HFFD in rats

**Figure 1:**
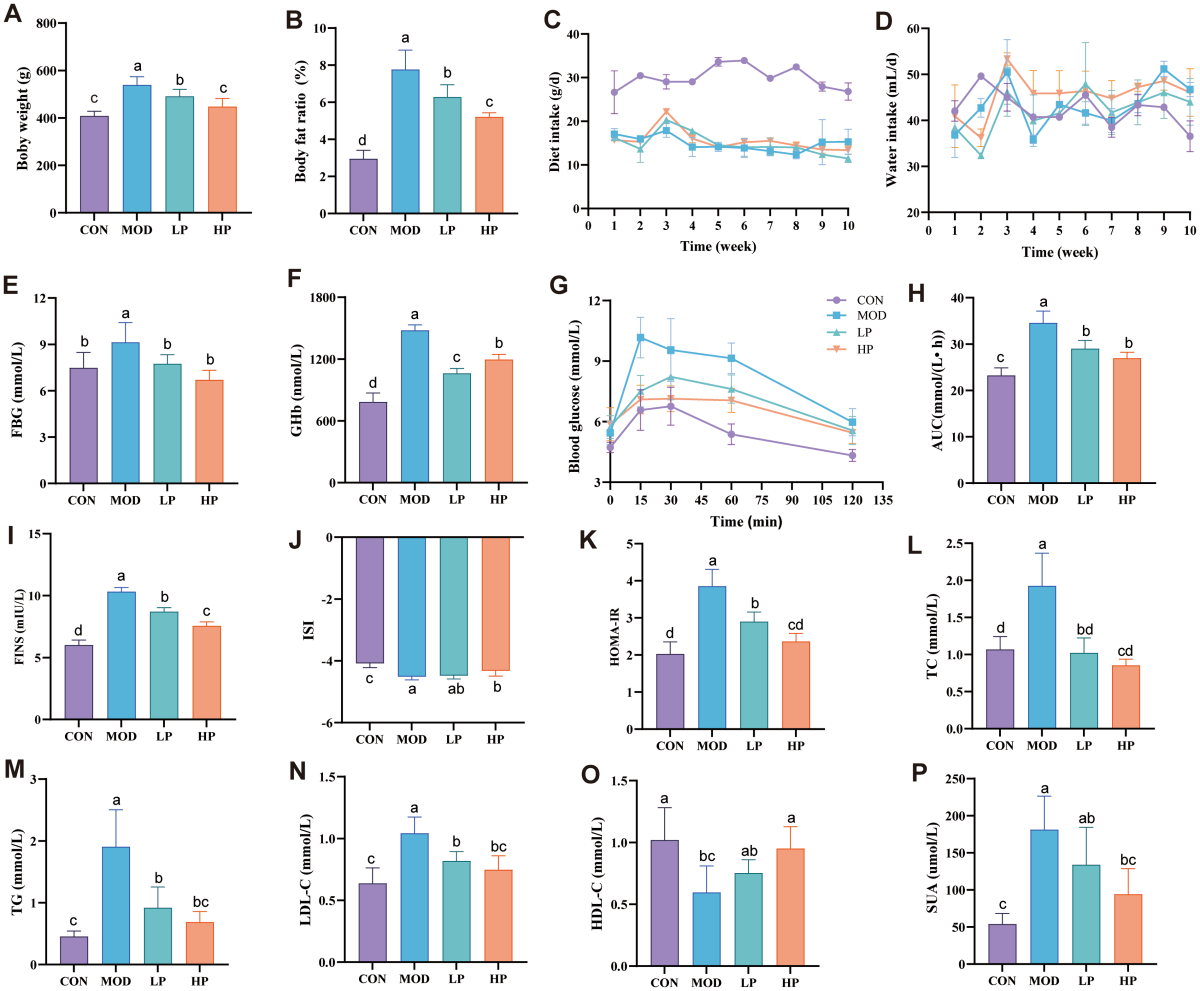
PXJ improves HFFD-induced metabolic dysregulation in rats. **(A,B)** Body weight and body fat percentage. **(C,D)** Daily food and water intake during the experimental period. **(E,F)** Serum FBG and GHb levels across groups. **(G)** Blood glucose determined at 0, 15, 30, 60, and 120 min after glucose administration. **(H)** AUC of oral glucose tolerance test results. **(I–P)** Serum FINS, ISI, HOMA-IR, TC, TG, LDL-C, HDL-C, SUA levels across groups. Data were expressed as the mean ± SEM from *n* = 8 rats per group. Different letters (a, b, c, d) indicate significant differences (*p* < 0.05); bars sharing the same letter are not significantly different.

PXJ significantly improved the weight gain and fat accumulation caused by HFFD in a dose-dependent manner. After 10 weeks of HFFD feeding, the body weight and body fat percentage of the MOD group were significantly higher than those of the CON group (*p* < 0.001). The improvement effects of PXJ on body weight and body fat were dose-dependent, with the HP group showing the most significant reduction in body weight and body fat (*p* < 0.001) (Figures 1A,B). The reduction in body weight and fat mass was independent of food or water intake, as no significant differences were observed in these measures among HFFD-fed rats (Figures 1C,D).

PXJ improved obesity while also reducing serum levels of blood glucose, lipids, and serum uric acid. More specifically, PXJ significantly reduced HFFD-induced hyperglycemia and improved insulin levels. HFFD-induced both fasting hyperglycemia and sustained elevation of GHb levels (*p* < 0.01, *p* < 0.001) in rats, which were reduced dose-dependently in the LP and HP groups (Figures 1E,F). PXJ also improved glucose metabolic capacity, as demonstrated by the OGTT results (Figures 1G,H). Meanwhile, we also monitored insulin levels, which are closely associated with glucose regulation. The MOD group exhibited significantly aggravated insulin resistance, specifically manifested by elevated FINS levels and HOMA-IR (*p* < 0.001), as well as reduced ISI (*p* < 0.001). PXJ ameliorated these abnormalities, with a more pronounced effect observed in the HP group (Figures 1I–K).

The HFFD also induced abnormal blood lipid profiles in rats, characterized by elevated TC, TG, and LDL-C levels (*p* < 0.001), along with reduced HDL-C levels (*p* < 0.01). PXJ reduced blood lipid levels, and more notably, the HP group showed levels comparable to those in the CON group (Figures 1L–O). This demonstrates that early PXJ intervention effectively improves lipid metabolism.

PXJ not only reduced blood glucose and lipid levels but also significantly lowered SUA levels. We observed markedly elevated SUA in the MOD group (*p* < 0.001), while both LP and HP groups showed reduced SUA levels, with a more pronounced decrease in the HP group (*p* < 0.001) (Figure 1P).

### 3.2 HFFD-induces pathological damage and the ameliorative effects of PXJ

**Figure 2:**
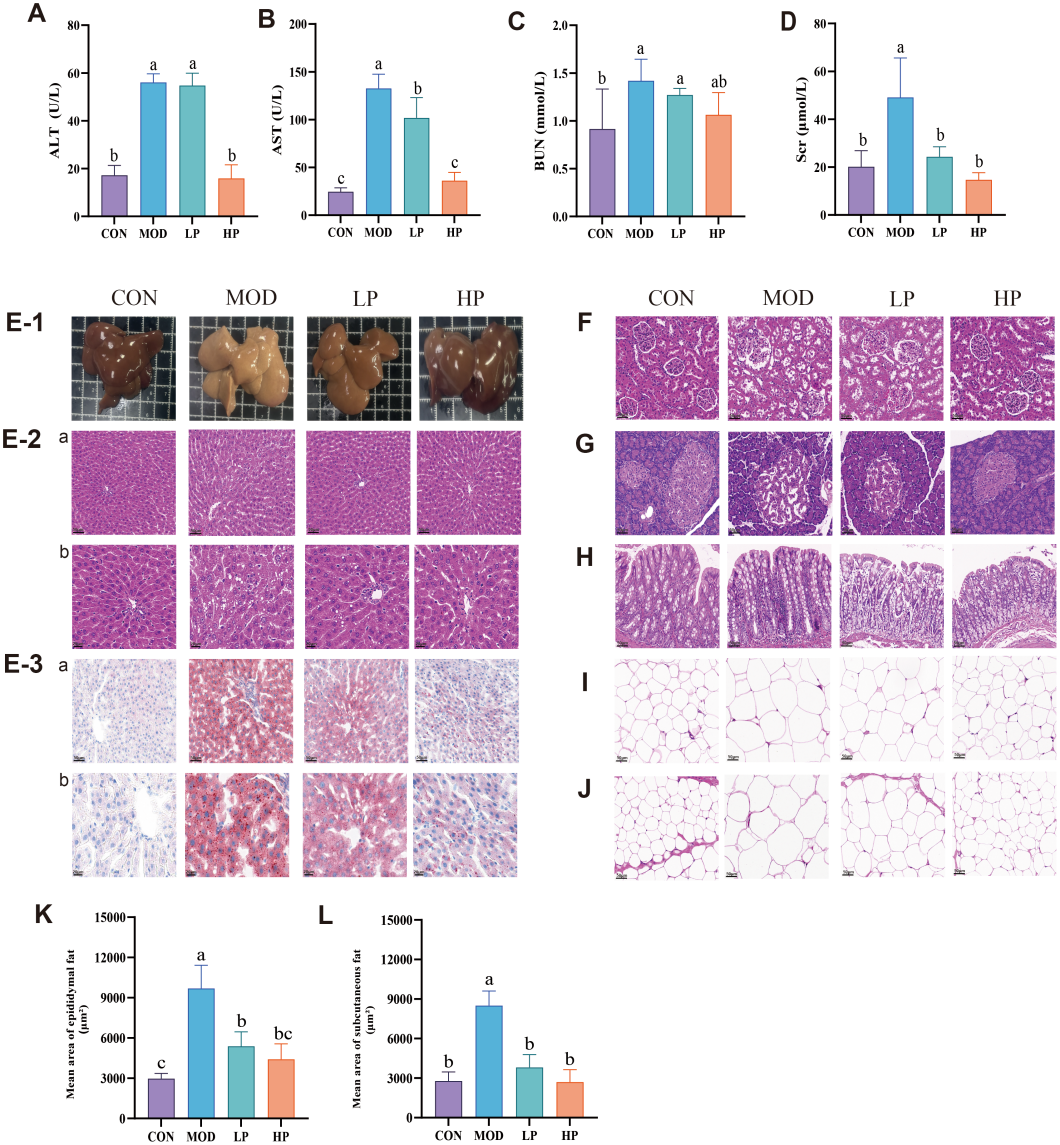
HFFD-induced pathological damage and the therapeutic effects of PXJ. **(A–D)** Serum ALT, AST, BUN and SCr levels across groups. **(E-1)** Fresh liver images, **(E-2a,b)** HE staining of the liver. **(E-3a,b)** ORO staining of the liver (*n* = 3, Scale bar, 50 μm and 20 μm). **(F–J)** HE staining of the kidney, pancreas, colon, epididymal adipose, subcutaneous adipose. (*n* = 3, Scale bar, 50 μm). **(K)** Epididymal fat average area. **(L)** Subcutaneous fat average area. Data were expressed as the mean ± SEM from *n* = 8 rats per group. Different letters (a, b, c) indicate significant differences (*p* < 0.05); bars sharing the same letter are not significantly different.

After 10 weeks of HFFD feeding, the levels of ALT, AST, BUN, and Scr in the MOD group were significantly higher than those in the CON group (Figures 2A–D), indicating that long-term HFFD caused significant impairment of liver and kidney function in the rats. However, early and sustained administration of PXJ ameliorated this phenomenon, particularly in the HP group.

Metabolic disorders are closely associated with pathological changes in the liver, kidney, pancreas, and intestines. However, directly observing such damage in clinical settings remains challenging. To address this, we investigated the impact of HFFD on the metabolic functions of major organs in rats using multiple pathological techniques.

**Figure 3:**
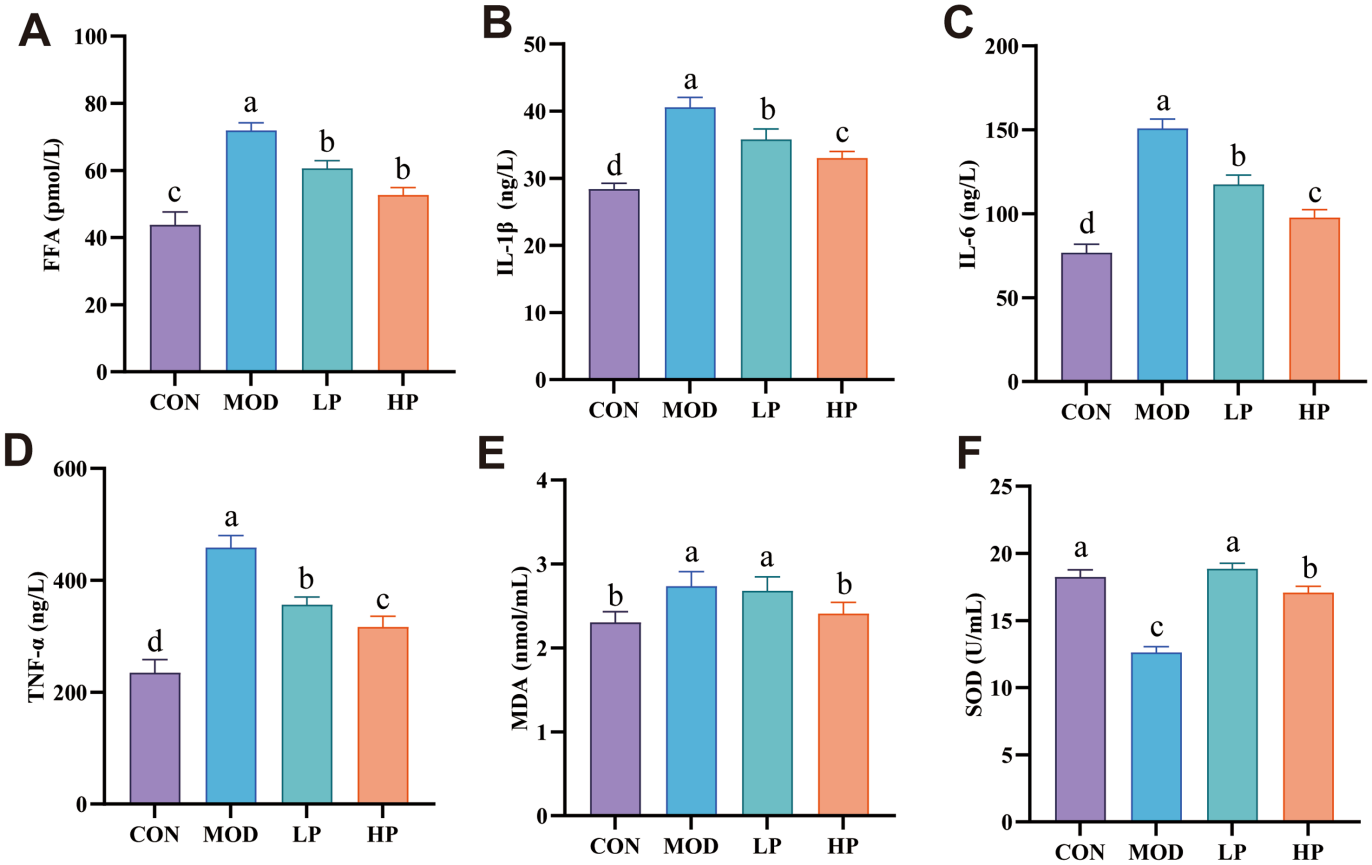
PXJ attenuates HFFD-induced inflammatory responses and oxidative stress. **(A)** Serum levels of FFA in each group. **(B–D)** Serum levels of IL-1*β*, IL-6, and TNF-*α* in each group. **(E,F)** Serum levels of MDA and SOD in each group. Data were expressed as the mean ± SEM from *n* = 8 rats per group. Different letters (a, b, c, d) indicate significant differences (*p* < 0.05); bars sharing the same letter are not significantly different.

At the termination of the experiment, we observed that the fresh liver in the MOD group appeared pale yellow or yellow-brown (Figure 2E-1), HE staining results (Figure 2E-2) revealed disorganization of the hepatic lobule structure, lipid vacuole accumulation in hepatocytes and inflammatory infiltration. Compared to the MOD group, PXJ gradually restored the liver’s macroscopic color to a dark red, resembling normal liver tissue. HE staining revealed a significant improvement in hepatic lobular structure, with reduced lipid vacuole accumulation and smaller vacuole sizes. Additionally, Oil Red O (ORO) staining intensity decreased, indicating reduced lipid droplet quantity within hepatocytes (Figure 2E-3). These results demonstrate that PXJ effectively improved liver lipid deposition and pathological damage induced by the HFFD, restoring the liver tissue structure to normal.

The kidney is one of the vital metabolic and excretory organs. Compared with the CON group, the MOD group exhibited pathological renal damage characterized by irregular glomerular structure, discontinuous renal tubular epithelial cells, and increased inflammatory cell aggregation (Figure 2F). PXJ restored regular glomerular architecture and reduced inflammatory infiltration, with more significant improvement observed in the HP group. The MOD group demonstrated widespread vacuolation and significant inflammatory infiltration in pancreatic tissues, potentially accounting for the disturbed glycemic regulation (Figure 2G). PXJ significantly alleviated these pathological changes, with the HP group showing more intact islet structures, tighter cellular arrangement, and substantially alleviated inflammatory infiltration. The colon in the MOD group showed pathological changes, including widespread destruction of crypt structures, further reduction in goblet cells, and inflammatory responses (Figure 2H). We observed improved crypt architecture and increased goblet cell numbers in both the LP and HP groups, along with a significant reduction in inflammatory responses.

Adipose tissue is a vital target organ for energy storage and metabolic regulation. From Figures 2I,J, it can be seen that the epididymal and subcutaneous adipocytes are significantly hypertrophic, indicating that a HFFD-induces noticeable fat accumulation. To further quantify the changes in adipocytes, the average area of epididymal and subcutaneous adipocytes was statistically analyzed (Figures 2K,L). The results showed that, in comparison with the CON group, the MOD group had larger epididymal and subcutaneous adipocyte areas. Importantly, HFFD-induced more significant fat accumulation in the epididymal adipose tissue. PXJ alleviated adipocyte hypertrophy, with the HP group showing the pronounced improvement (*p* < 0.001).

### 3.3 PXJ alleviates HFFD-induced free fatty acid accumulation, inflammatory response, and oxidative stress dysregulation

Based on changes in metabolic parameters, histopathological observations, and biochemical markers, the mechanisms underlying HFFD-induced metabolic disorders and the protective effects of PXJ intervention were further investigated. After 10 weeks of HFFD feeding, serum FFA levels were significantly increased, while PXJ effectively reduced serum FFA levels (*p* < 0.001) (Figure 3A). Meanwhile, we further observed that PXJ significantly attenuated HFFD-induced elevation of serum inflammatory cytokines, including IL-1β, IL-6, and TNF-*α* (*p* < 0.001) (Figures 3B–D).

Additionally, elevated MDA and reduced SOD activity were observed in the MOD group serum (*p* < 0.001). Importantly, PXJ demonstrated a dose-dependent improvement in oxidative stress parameters (Figures 3E,F).

### 3.4 PXJ altered the diversity and composition of the gut microbiota

**Figure 4:**
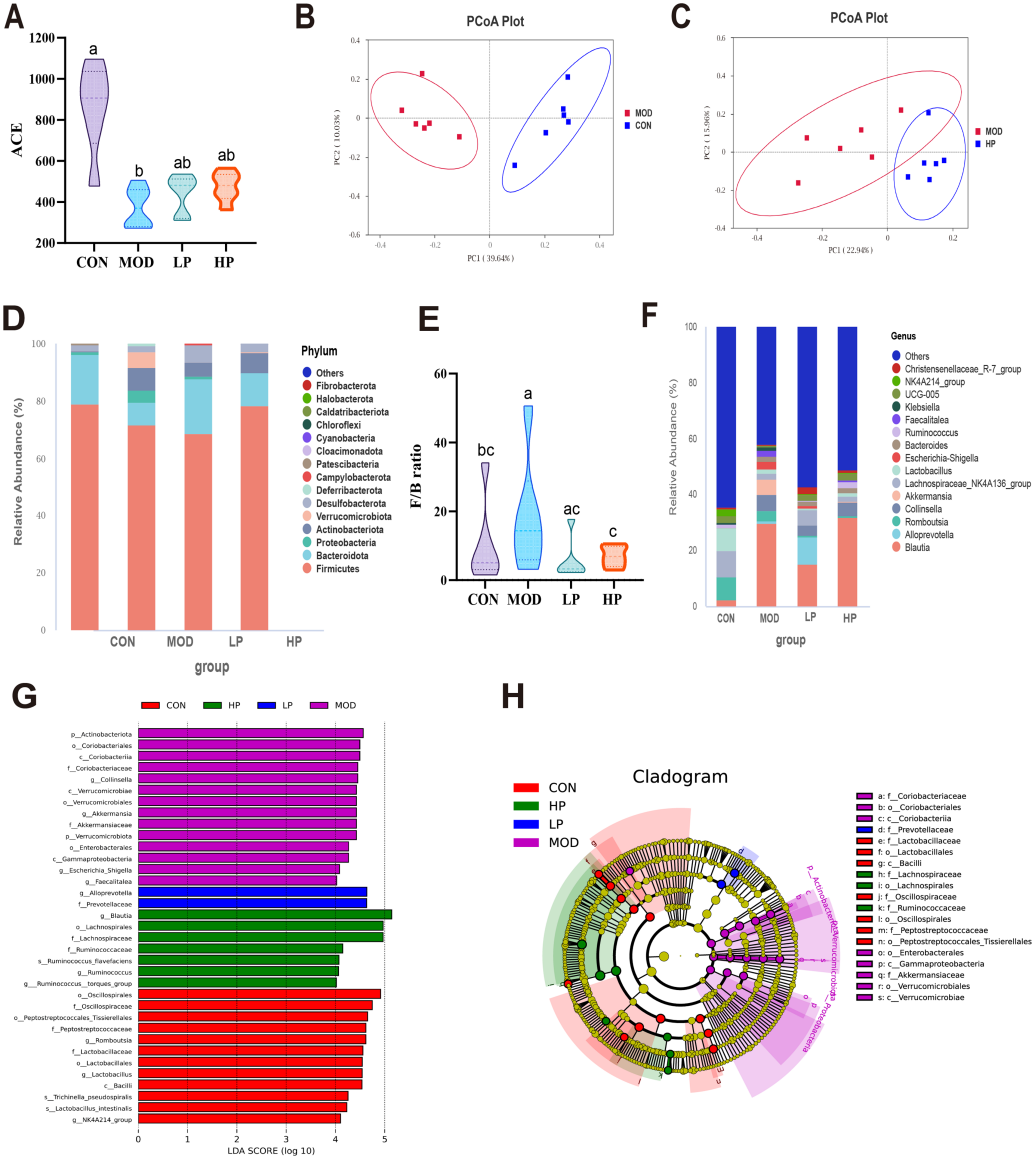
PXJ altered the composition and functionality of the gut microbiota within HFFD rats. **(A)** α diversity (ACE) of gut microbiota in rats. **(B,C)** β diversity, PCoA depicting clustering of the three groups. **(D)** Phylum-level composition of gut microbiota. **(E)** The ratio of Firmicutes/Bateroidota. **(F)** Genus-level composition of gut microbiota. **(G)** LDA score plot. (LDA score > 4 and *p*-value < 0.05, established through the Kruskal-Wallis test). **(H)** Cladogram generated from LEfSe analysis. Data were expressed as the mean ± SEM from *n* = 6 rats per group. Different letters (a, b, c) indicate significant differences (*p* < 0.05); bars sharing the same letter are not significantly different.

The impact of PXJ on gut microbiota in HFFD was analyzed using 16S rRNA sequencing technology. As illustrated in Figure 4A, the ACE index of gut microbiota in MOD group rats significantly decreased after HFFD intervention (*P* < 0.001). Moreover, the PCoA plot revealed significant variations in gut microbiota composition between the CON group and MOD group, as well as between the MOD group and HP group, as shown in Figures 4B,C.

At the phylum level, gut microbiota analysis identified *Firmicutes*, *Bacteroidota*, *Proteobacteria*, and *Actinobacteria* as the major microbial communities. Compared to the CON group, the MOD group exhibited a significant increase in the relative abundances of *Proteobacteria* and *Actinobacteriota*, accompanied by an elevated F/B ratio (*p* < 0.05) (Figures 4D,E). Subsequent analysis at the genus level further revealed the alterations in gut microbiota composition (Figure 4F). In the MOD group, probiotics such as *Ruminococcus* and *Lachnospiraceae_NK4A136_group* were significantly reduced, while pathogenic bacteria like *Collinsella* and *Escherichia-Shigella* increased. We found that early and sustained administration of PXJ effectively mitigated HFFD-induced gut microbiota dysbiosis at both the phylum and genus levels. For instance, the LP and HP groups exhibited a decreased F/B ratio and elevated *Alloprevotella* levels.

The LDA plots showed the specific bacteria from phylum to genus level. Our re-sults showed that 35 different bacteria were sifted in the four groups, with 12, 14, 2 and 7 significant differences in the CON, MOD, LP and HP groups (Figures 4G,H). The MOD group exhibited the highest number of differentially gut bacteria, including the family *Akkermansiaceae* and its affiliated genus *Akkermansia*, along with the genus *Escherichia_Shigella* in class *γ-Proteobacteria*, totaling 14 significantly enriched microbial taxa. The LP group was characterized by the genus *Alloprevotella* and the family *Prevotellaceae*, and the HP group was predominantly represented by the family *Lachnospiraceae* and family *Ruminococcaceae*. This indicates that interventions in the LP and HP groups can partially improve the gut microbiota structure and potentially provide metabolic protection.

### 3.5 PXJ modulates the plasma metabolome in HFFD-fed rats

**Figure 5:**
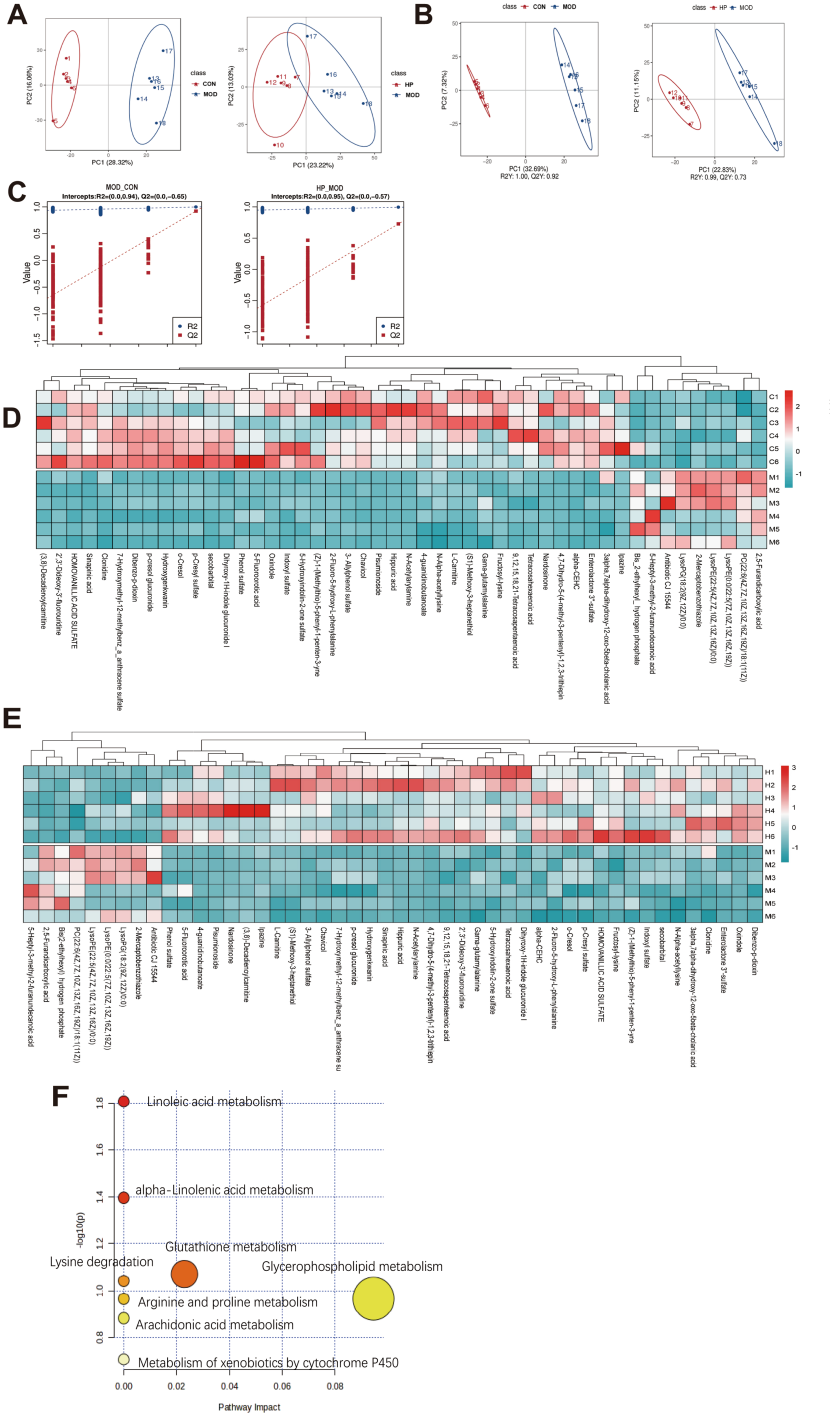
PXJ modulated the plasma metabolic profile in HFFD rats. **(A)** PCA score plot. **(B)** PLS-DA score scatter plot. **(C)** PLS-DA permutation test plot. **(D,E)** Hierarchical clustering heat map of metabolites in the CON, MOD and HP groups. **(F)** Results of the pathway enrichment analysis of differentially accumulated metabolites.

Metabolomic analysis revealed noteworthy differences in plasma metabolites between CON, MOD and HP groups, demonstrating that plasma metabolites may mediate the process of rat metabolism. The PLS-DA model displayed distinct clustering among the three groups, which was confirmed by a permutation test (Figures 5A–C).

We identified 79 differential metabolites between the CON and MOD groups, as well as between the MOD and HP groups, based on VIP > 1 and *p* < 0.05 (Supplementary Table S2). Utilizing the HMDB database for further annotation, we successfully identified 48 biomarkers. Among these, lipids and lipid-like molecules, as well as organic acids and derivatives, were the predominant categories. The comparative analysis showed that, compared to CON, MOD exhibited 9 upregulated and 39 downregulated metabolites, while relative to HP, MOD displayed 39 upregulated and 9 downregulated metabolites. As shown in the heatmap, the metabolism of lipids and lipid-like molecules, as well as organic acids and their derivatives, showed significant reversal in the HP group, suggesting that these metabolites may participate in the metabolic regulation mediated by PXJ (Figures 5D,E and Supplementary Table S3).

Furthermore, pathway analysis using MetaboAnalyst 6.0 (25)found eight metabolic pathways potentially regulated by PXJ, such as glycerophospholipid metabolism, glutathione metabolism, *α*-linolenic acid metabolism, linoleic acid metabolism, and arachidonic acid metabolism (Figure 5F).

### 3.6 Correlations between gut microbiota, metabolites, and inflammatory cytokines, oxidative stress markers

**Figure 6:**
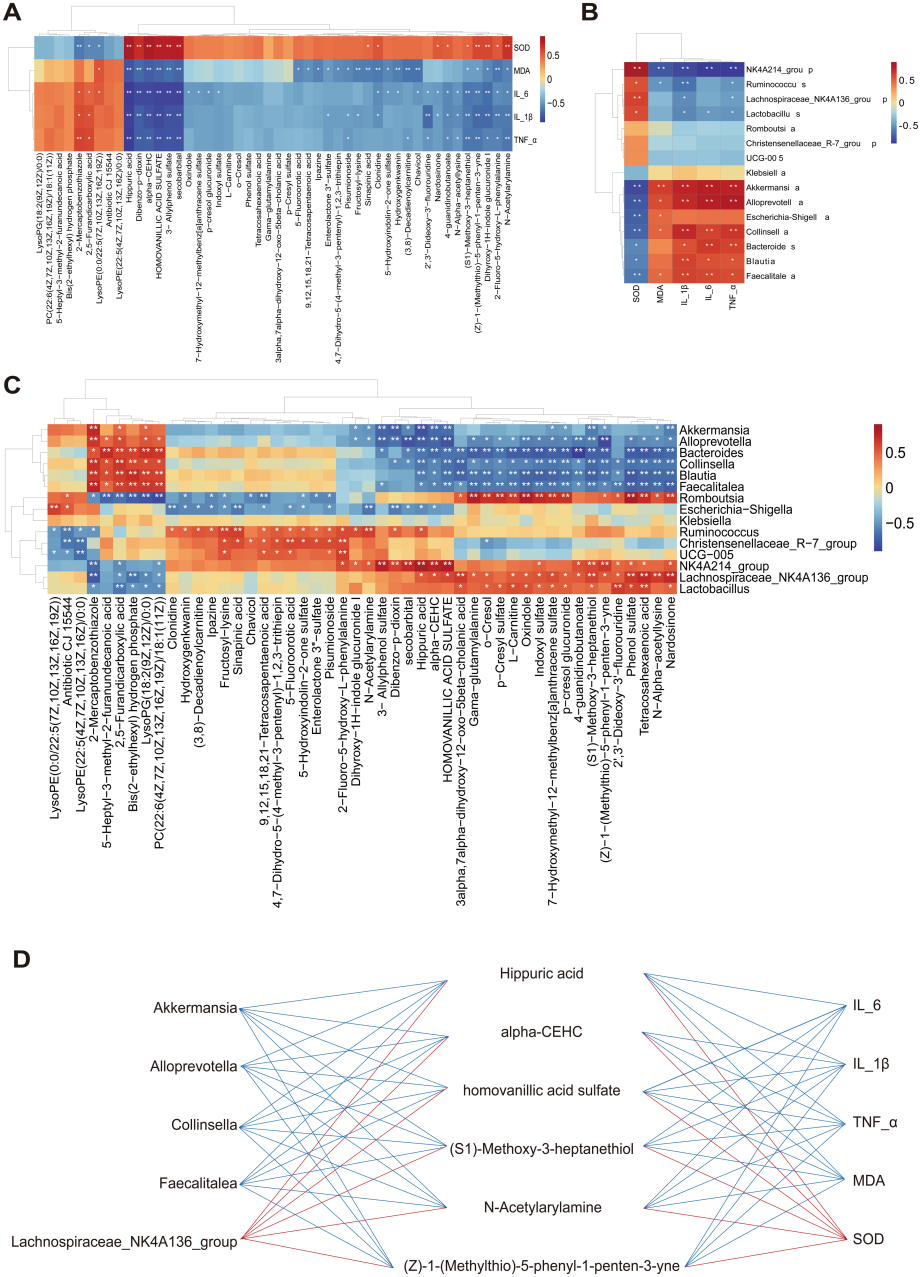
Correlations between gut microbiota, metabolites, and inflammatory cytokines, oxidative stress markers. **(A)** Correlation analysis between inflammatory cytokines, oxidative stress markers and gut microbiota at the genus level. **(B)** Correlation analysis between inflammatory cytokines, oxidative stress markers and metabolites. **(C)** Correlation analysis between the gut microbiota genus and metabolites ^*^p < 0.05 and ^**^p < 0.01. **(D)** The interactions between significantly correlated pairs of microbiota-metabolite-inflammatory cytokine and oxidative stress markers. The red (blue) line represented a positive correlation (negative correlation).

The Spearman correlation analysis was performed to potential relationships among gut microbiota at the genus level, metabolites, and inflammatory cytokines, oxidative stress markers. As shown in Figure 6A, *Akkermansia*, *Alloprevotella*, *Faecalitalea*, and *Collinsella* exhibited a positive correlation with inflammatory cytokines but a negative correlation with SOD, whereas *NK4A214_group* showed the completely opposite pattern.

The associated relationship between inflammatory cytokines, oxidative stress markers and metabolites was conducted and is displayed in Figure 6B. In general, organic acid metabolites such as hippuric acid and homovanillic acid sulfate were negatively correlated with inflammatory factors and MDA, while showing a significant positive correlation with SOD. The same trend was observed in the benzene-class metabolites (Z)-1-(Methylthio)-5-phenyl-1-penten-3-yne and N-Acetylarylamine, as well as the organic oxygen compound (S1)-Methoxy-3-heptanethiol.

Subsequently, correlation analysis of gut microbiota and metabolites demonstrated a stronger relationship between them (Figure 6C). The lipid metabolites, 5-Heptyl-3-methyl-2-furanundecanoic acid and PC (22:6(4Z,7Z,10Z,13Z,16Z,19Z)/18:1(11Z)) showed a negative correlation with *Romboutsia* and *Lachnospiraceae_NK4A214_group*, but a positive correlation with *Alloprevotella* and *Collinsella*. Interestingly, we observed that the benzene-class metabolites (Z)-1-(Methylthio)-5-phenyl-1-penten-3-yne and N-Acetylarylamine, as well as organic acids and their derivatives such as hippuric acid and homovanillic acid sulfate, exhibited completely opposite correlations with the aforementioned gut microbiota.

Furthermore, we integrated the differentially expressed metabolites, gut microbiota, and inflammatory and oxidative stress identified in Figures 6A–C factors to investigate how early and sustained PXJ intervention in HFFD rats influenced the relationships among these three components (Figure 6D). It was found that the six metabolites such as hippuric acid, alpha-CEHC, homovanillic acid sulfate and (S1)-Methoxy-3-heptanethiol were positively correlated with the *Lachnospiraceae_NK4A214_group*, however negatively correlated with *Akkermansia*, *Alloprevotella*, *Collinsella*, and *Faecalitalea*. Meanwhile, it was found that these six metabolites were positively correlated with SOD but negatively correlated with IL-6, IL-1β, TNF-*α* and MDA. This suggests a complex and close interrelationship among gut microbiota, metabolites, and inflammatory, oxidative stress factors.

## 4 Discussion

Long-term intake of foods high in saturated fats and beverages containing fructose is one of the primary causes of metabolic disorders (26, 27). These metabolic disturbances frequently co-occur and form a vicious cycle (28). Previous studies have confirmed that HFFD can lead to multiple metabolic imbalances of glycolipids and serum uric acid, but there are relatively few studies that jointly focus on the metabolic disorders of the three. Under the current dietary pattern, the metabolic disorders of glycolipids and serum uric acid are more in line with the current disease situation and also conform to the evolution law of the disease from the perspective of pathogenesis. The rat model in our study was fed with HFFD, which closely mimics unhealthy dietary patterns in modern daily life, such as high-calorie Western diets (29–31). The elevated blood indicators, such as blood lipid and blood glucose, as well as pathological organ alterations observed in the MOD group are consistent with the characteristic manifestations of metabolic disorder models (32–34). Early and consistent administration of PXJ can significantly improve metabolic imbalance in rats, manifested by reductions in blood glucose and lipid levels, synchronized improvements in pathological morphology of metabolism-related organs and tissues such as the liver, kidney, pancreas, and adipose tissue, as well as certain protective effects on hepatorenal function. Previous studies have found that Helixor M, an extract from *Viscum album L.* growing on the apple tree, showed activity against methotrexate-induced acute oxidative stress and nephrotoxicity in rats (35). Additionally, *Viscum articulatum* Burm. f., it was shown that mistletoe had a significant effect on the urine excretion volume (36, 37). Nevertheless, previous studies have not focused on uric acid levels. Our research found that PXJ not only exhibits renal protective effects but also reduces serum uric acid levels in rats with metabolic disorders. This discovery may reveal a novel therapeutic property of the mistletoe species.

Maintaining the normal morphology of metabolic organs is essential for the body’s long-term metabolic homeostasis (38, 39). It was observed that PXJ not only improved main metabolic biochemical parameters, but also concurrently alleviated internal organ damage, as systematically demonstrated through histopathological examination of tissue sections. The liver serves as the primary organ responsible for regulating both blood glucose and lipid metabolism, with intricate interplay between these two metabolic pathways (40). PXJ effectively reduces hepatic lipid accumulation and promotes the repair of lobular hepatocytes, this might be one of the mechanisms by which it improves glycolipid metabolism. In addition, this cooperative effect finds expression in the restoration of glomeruli, optimization of kidney function, and decline in serum uric acid. Histopathological analysis of pancreatic tissue similarly revealed attenuated inflammatory infiltration and well-preserved islet cell morphology, both of which are crucial for maintaining glucose homeostasis. The chronic inflammation in metabolic disorders is termed metabolic inflammation and is characterized by low-grade local or systemic inflammatory responses (41, 42). This accounts for the observed rise in serum inflammatory, oxidative stress markers in the MOD group, concurrent with pronounced inflammatory pathology in metabolic organs. To elaborate further, the disease manifestations of metabolic disorders in the results establish correlations between measurable biomarker factors and occult visceral pathological damage, facilitating the clinical utilization of blood indices to reflect the intrinsic disease status of patients with metabolic dysregulation, particularly the severity of visceral inflammation.

The composition and function of the gut microbiota are dynamic and influenced by dietary patterns, thereby altering host metabolism and the incidence of metabolic disorders (43, 44). Consequently, modulating the types or proportions of orally ingested foods can affect the balance and functionality of gut microbiota, representing a direct approach to ameliorating metabolic disorders (44, 45). We confirmed that early oral administration of PXJ effectively ameliorates HFFD-induced metabolic disorders, associated with improved gut microbiota, modulated metabolites, and reduced inflammatory oxidative stress. These effects exhibit sustained durability, with the complex interplay between mechanisms. Lipids and other energy substrates affect the gut microbiota both as substrates for bacterial metabolic processes and by inhibiting bacterial growth by toxic influence (43). Through heatmap analysis, we found that PXJ may participate in modulating the gut microbiota, increasing microbial diversity, while upregulating beneficial bacteria, such as, *Akkermansia*, *Lachnospiraceae_NK4A214_group.* Their colonization dynamics balance depends on dietary fats and fibers, while collectively mediating short-chain fatty acid (SCFA) biosynthesis to orchestrate inflammatory responses and energy metabolism homeostasis (46, 47). Moreover, the down regulated LysoPG (18:2) in PXJ-treated groups (Figure 5E) not only modulates lipid metabolism but also reduces glomerular and tubular injuries in renal tissues (48). Meanwhile, PXJ may modulate the gut microbiota to promote the production of hippuric acid, a metabolite that facilitates uric acid excretion (49). This suggests that the metabolic improvements induced by PXJ may not stem from a single target, but rather involve an integrated microbiota-metabolism-cytokines axis. The dynamic equilibrium of gut microbiota shows a correlation with the progression of metabolic diseases. Maintaining metabolic balance through modulation of gut microbiota homeostasis may represent a promising direction for future research. Our correlation analysis further revealed that organic acids and their derivatives, as well as benzene compounds, are closely related to the gut microbiota. This may be related to the parasitism of PXJ on the tea plant. PXJ is rich in polyphenols, flavonoids, alkaloids, and other bioactive components (14), which may contribute to its ability to improve gut microbiota and their metabolites, thereby reducing blood sugar and lipid levels. Based on the pwise correlation analysis of “microbiota – metabolites – cytokines,” even if we only selected those with significant correlations to construct the “microbiota – metabolites – cytokines” association network diagram (Figure 6D), it can still indicate the complexity of the mechanism of sugar and lipid metabolism disorders. Another study on the improvement of NASH by green tea also proved this point, which was achieved by the dynamic modulation of 122 pivotal metabolites spanning amino acid pathways, lipid species, and their interconnected metabolic networks (50). These interconnected factors collectively participate in the regulatory process, and network complexity escalates progressively with the inclusion of each additional regulatory factor. These findings suggest that metabolic disorders induced by HFFD diets exhibit remarkable complexity, and combating such complexity may require equally sophisticated network-based intervention strategies, among which dietary interventions could serve as one of the safer, more natural, and less invasive alternative. Recently, one study has also found that dietary intervention can better improves the disorders caused by high-calorie diets than microbiota transplantations (51). During the HFFD-induced metabolic disorder process, early and consistent oral administration of PXJ aqueous extract demonstrated its multitarget characteristics, which was associated with restored gut microbiota balance, improved metabolism. This intervention effectively counteracted inflammatory and oxidative stress responses, exhibiting significant therapeutic potential in systemic metabolic regulation.

Furthermore, previous studies on PXJ primarily concentrated on alcohol-extracted compounds as the primary research focus (14, 52). The aqueous extraction method facilitates high yields of total free amino acids and tea polysaccharides from PXJ (53). Our study adopted the water extraction method, confirming that PXJ can improve the state of metabolic disorders in the long term, and this method is more applicable to daily life. Analysis via HS-SPME and GC–MS revealed that PXJ exhibits a clean, fresh aroma with woody and nutty notes accompanied by a subtle floral undertone, and sensory evaluation further confirmed its acceptable taste and aroma (12). These findings collectively suggest that PXJ, considering both its functional properties and sensory acceptability, holds promise as a novel functional tea beverage for daily consumption. These findings provide a novel intervention strategy for the early prevention and long-term management of metabolic disorders. While this study provides insights into the effects of PXJ, several limitations should be addressed in future work. First, the absence of a normal-diet combined with PXJ group precludes distinction between PXJ’s specific antagonistic effects on HFFD and its intrinsic pharmacological properties. Second, although 16S rRNA sequencing and metabolomics revealed microbiota-metabolite-inflammation correlations, causal relationships remain unverified by interventional experiments (e.g., fecal microbiota transplantation). Additionally, single-timepoint sampling at intervention endpoint limited dynamic observation of metabolic trajectories. Future studies should incorporate longitudinal sampling, germ-free models, and compound isolation to elucidate the precise mechanistic pathways.

## 5 Conclusion

The HFFD effectively mimics contemporary dietary patterns, with its model characteristics showing high consistency with the phenotypic manifestations of multiple metabolic disorders. When administered concomitantly, PXJ effectively reduced various metabolic disturbances, including impaired glucose, lipid homeostasis and serum uric acid dysregulation, while providing organ-protective effects. Notably, correlation network analysis reveals significant interactions among gut microbiota, metabolites, and inflammatory markers, suggesting that PXJ may exert systemic effects associated with the “microbiota-metabolism-cytokines” axis.

Through systematic biochemical profiling and histopathological examination, we validated PXJ’s concurrent metabolic-modulating and organ-preserving properties. It is the first to combine 16S rRNA sequencing with untargeted metabolomics to preliminarily elucidate the potential mechanisms of PXJ. These findings offer new evidence for the potential of PXJ as a functional tea beverage that can intervene early in metabolic dysregulation.

## Data Availability

The original contributions presented in the study are publicly available. This data can be found here: https://www.ncbi.nlm.nih.gov/bioproject/PRJNA1320938/ and https://www.ebi.ac.uk/metabolights/MTBLS12949.

## Footnotes

^1^ https://www.arb-silva.de/

^2^ https://www.metaboanalyst.ca/

^3^ https://www.bioincloud.tech

## Glossary

HFFD: high-fat and high-fructose diet
PXJ: Pangxiejiao (*Viscum articulatum* Burm.f.)
CON: Control group
MOD: HFFD group
LP: Low-dose group
HP: High-dose group
OGTT: Oral Glucose Tolerance Test
FBG: Fasting blood glucose
TC: total cholesterol
TG: triglycerides
HDL-C: high-density lipoprotein cholesterol
LDL-C: low-density lipoprotein cholesterol
SUA: serum uric acid
ALT: alanine aminotransferase
AST: aspartate aminotransferase
BUN: blood urea nitrogen
Scr: serum creatinine
GHb: Glycated hemoglobin
FINS: fasting insulin
FFA: free fatty acids
IL-1β: interleukin-1β
IL-6: interleukin-6
TNF-α: tumor necrosis factor-α
MDA: malondialdehyde
SOD: superoxide dismutase
HOMA-IR: homeostasis model assessment of insulin resistance
ISI: insulin sensitivity index
