# Supplementary material for: An analysis of the intestinal microbiome combined with metabolomics to explore the mechanism of how Pangxiejiao tea improves metabolic disorders in HFFD-treated rats
Source: Front Nutr. 2025 Sep 17;12:1653855. doi: 10.3389/fnut.2025.1653855 (PMC12483856; doi:10.3389/fnut.2025.1653855)
Supplement: Supplementary file 1 [file Table_1.DOCX]

**Table S1.** Composition of high-fat die.

| Ingredient | gm | kcal |
| --- | --- | --- |
| Casein | 200 | 800 |
| L-Gystine | 3 | 12 |
| Corn Starch | 72.8 | 291.2 |
| Maltodextrin | 100 | 400 |
| Sucrose | 176.8 | 707.2 |
| Cellulose | 50 | 0 |
| Soybean Oil | 25 | 225 |
| Lard | 177.5 | 1597.5 |
| Mineral Mix S10026B | 50 | 0 |
| Vitamin Mix V10001C | 1 | 4 |
| Choline Bitartrate | 2 | 0 |
| Total | 858.15 | 4036.9 |

The basal diet for rat feeding was provided by the Animal Center of Guangdong Pharmaceutical University. The raw material composition includes corn, soybean meal, fish meal, wheat flour, yeast powder, vegetable oil, salt, multivitamins, and mineral elements, among others. The specific ingredient ratio involves technical processes and cannot be disclosed, but the functional ratio is provided.

**Table S2.** The reversed differential metabolites.

| NO. | Name | Formula | HMDB_ID | m/z | RT (min) | MOD vs. CON | HP vs. MOD | Adduct |
| --- | --- | --- | --- | --- | --- | --- | --- | --- |
| 1 | 5-Heptyl-3-methyl-2-furanundecanoic acid | C_23_H_40_O_3_ | HMDB0112075 | 363.29 | 7.78 | ↑^**^ | ↓^*^ | [M-H]- |
| 2 | Antibiotic CJ 15544 | C_25_H_34_O_6_ | HMDB0033080 | 431.24 | 10.61 | ↑^**^ | ↓^*^ | [M+H]+ |
| 3 | 2,5-Furandicarboxylic acid | C_6_H_4_O_5_ | HMDB0004812 | 139 | 6.49 | ↑^***^ | ↓^***^ | [M+H-H2O]+ |
| 4 | LysoPE(0:0/22:5(7Z,10Z,13Z,16Z,19Z)) | C_27_H_46_NO_7_P | HMDB0011495 | 528.31 | 9.12 | ↑^**^ | ↓^**^ | [M+H]+ |
| 5 | LysoPE(22:5(4Z,7Z,10Z,13Z,16Z)/0:0) | C_27_H_46_NO_7_P | HMDB0011524 | 526.29 | 9.12 | ↑^**^ | ↓^*^ | [M-H]- |
| 6 | 2-Mercaptobenzothiazole | C_7_H_5_NS_2_ | HMDB0030524 | 165.98 | 6.01 | ↑^**^ | ↓^*^ | [M-H]- |
| 7 | PC(22:6(4Z,7Z,10Z,13Z,16Z,19Z)  /18:1(11Z)) | C_48_H_82_NO_8_P | HMDB0008728 | 854.57 | 10.54 | ↑^*^ | ↓^*^ | [M+Na]+ |
| 8 | LysoPG(18:2(9Z,12Z)/0:0) | C_24_H_45_O_9_P | HMDB0240600 | 507.27 | 8.96 | ↑^**^ | ↓^*^ | [M-H]- |
| 9 | Bis(2-ethylhexyl) hydrogen phosphate | C_16_H_35_O_4_P | HMDB0249242 | 321.22 | 8.64 | ↑^**^ | ↓^***^ | [M-H]- |
| 10 | 9,12,15,18,21-Tetracosapentaenoic acid | C_24_H_38_O_2_ | HMDB0247624 | 357.28 | 10.9 | ↓^***^ | ↑^***^ | [M-H]- |
| 11 | L-Carnitine | C_7_H_16_NO3 | HMDB0000062 | 162.11 | 1.39 | ↓^***^ | ↑^**^ | [M+H]+ |
| 12 | (3,8)-Decadienoylcarnitine | C_17_H_29_NO_4_ | HMDB0241094 | 312.22 | 6.06 | ↓^**^ | ↑^*^ | [M+H]+ |
| 13 | Gama-glutamylalanine | C_8_H_14_N_2_O_5_ | HMDB0006248 | 219.1 | 1.85 | ↓^*^ | ↑^**^ | [M+H]+ |
| 14 | (S1)-Methoxy-3-heptanethiol | C_8_H_18_OS | HMDB0032380 | 163.12 | 1.39 | ↓^***^ | ↑^**^ | [M+H]+ |
| 15 | N-Alpha-acetyllysine | C_8_H_16_N_2_O_3_ | HMDB0000446 | 189.12 | 1.49 | ↓^**^ | ↑^*^ | [M+H]+ |
| 16 | Oxindole | C_8_H_7_NO | HMDB0061918 | 134.06 | 5.69 | ↓^**^ | ↑^***^ | [M+H]+ |
| 17 | 3alpha,7alpha-dihydroxy-12-oxo-5beta-cholanic acid | C_24_H_38_O_5_ | HMDB0000400 | 407.28 | 6.69 | ↓^*^ | ↑^**^ | [M+H]+ |
| 18 | Fructosyl-lysine | C_12_H_24_N_2_O_7_ | HMDB0034879 | 309.17 | 1.22 | ↓^**^ | ↑^***^ | [M+H]+ |
| 19 | Clonidine | C_9_H_9_C_l2_N_3_ | HMDB0014714 | 228.01 | 11.99 | ↓^***^ | ↑^*^ | [M-H]- |
| 20 | Chavicol | C_9_H_10_O | HMDB0034107 | 133.07 | 5.83 | ↓^***^ | ↑^***^ | [M-H]- |
| 21 | Tetracosahexaenoic acid | C_24_H_36_O_2_ | HMDB0002007 | 355.26 | 10.42 | ↓^***^ | ↑^*^ | [M-H]- |
| 22 | HOMOVANILLIC ACID SULFATE | C_9_H_10_O_7_S | HMDB0011719 | 261.01 | 5.15 | ↓^***^ | ↑^**^ | [M-H]- |
| 23 | Indoxyl sulfate | C_8_H_7_NO_4_S | HMDB0000682 | 212 | 5.25 | ↓^***^ | ↑^**^ | [M-H]- |
| 24 | Sinapinic acid | C_11_H_12_O_5_ | HMDB0034069 | 223.06 | 5.85 | ↓^***^ | ↑^**^ | [M-H]- |
| 25 | Dibenzo-p-dioxin | C_12_H_8_O_2_ | HMDB0255966 | 185.06 | 5.6 | ↓^***^ | ↑^**^ | [M+H]+ |
| 26 | Nardosinone | C_15_H_22_O_3_ | HMDB0255460 | 273.15 | 6.33 | ↓^**^ | ↑^*^ | [M+Na]+ |
| 27 | 2',3'-Dideoxy-3'-fluorouridine | C_9_H_11_FN_2_O_4_ | HMDB0245543 | 231.08 | 5.84 | ↓^***^ | ↑^***^ | [M+H]+ |
| 28 | p-cresol glucuronide | C_13_H_16_O_7_ | HMDB0011686 | 283.08 | 5.61 | ↓^***^ | ↑^**^ | [M-H]- |
| 29 | p-Cresyl sulfate | C_7_H_8_O_4_S | HMDB0011635 | 187.01 | 6.73 | ↓^**^ | ↑^*^ | [M-H]- |
| 30 | 5-Hydroxyindolin-2-one sulfate | C_8_H_7_NO_5_S | HMDB0304927 | 228 | 5.12 | ↓^***^ | ↑^**^ | [M-H]- |
| 31 | Hydroxygenkwanin | C_16_H_12_O_6_ | HMDB0037339 | 323.05 | 5.61 | ↓^***^ | ↑^*^ | [M+Na]+ |
| 32 | 7-Hydroxymethyl-12-methylbenz[a]anthracene sulfate | C_20_H_16_O_4_S | HMDB0060420 | 351.07 | 5.61 | ↓^***^ | ↑^*^ | [M-H]- |
| 33 | Dihyroxy-1H-indole glucuronide I | C_14_H_15_NO_8_ | HMDB0059997 | 324.07 | 5.19 | ↓^***^ | ↑^**^ | [M-H]- |
| 34 | 4-guanidinobutanoate | C_5_H_11_N_3_O_2_ | HMDB0003464 | 146.09 | 1.81 | ↓^*^ | ↑^**^ | [M+H]+ |
| 35 | o-Cresol | C_7_H_8_O | HMDB0002055 | 107.05 | 5.52 | ↓^***^ | ↑^*^ | [M-H]- |
| 36 | secobarbital | C_12_H_18_N_2_O_3_ | HMDB0014562 | 239.14 | 5.53 | ↓^***^ | ↑^**^ | [M+H]+ |
| 37 | Phenol sulfate | C_6_H_6_O_4_S | HMDB0060015 | 172.99 | 5.2 | ↓^**^ | ↑^*^ | [M-H]- |
| 38 | alpha-CEHC | C_16_H_22_O_4_ | HMDB0001518 | 277.14 | 6.31 | ↓^***^ | ↑^**^ | [M-H]- |
| 39 | 5-Fluoroorotic acid | C_5_H_3_FN_2_O_4_ | HMDB0246795 | 173 | 5.19 | ↓^**^ | ↑^*^ | [M-H]- |
| 40 | (Z)-1-(Methylthio)-5-phenyl-1-penten-3-yne | C12H12S | HMDB0032688 | 187.06 | 5.89 | ↓^**^ | ↑^*^ | [M-H]- |
| 41 | 2-Fluoro-5-hydroxy-L-phenylalanine | C_9_H_10_FNO_3_ | HMDB0244653 | 200.07 | 5.09 | ↓^***^ | ↑^**^ | [M+H]+ |
| 42 | 3- Allylphenol sulfate | C_9_H_10_O_4_S | HMDB0304933 | 213.02 | 5.83 | ↓^***^ | ↑^***^ | [M-H]- |
| 43 | Hippuric acid | C_9_H_9_NO_3_ | HMDB0000714 | 178.05 | 5.43 | ↓^***^ | ↑^***^ | [M-H]- |
| 44 | N-Acetylarylamine | C_8_H_9_NO | HMDB0001250 | 134.06 | 5.43 | ↓^***^ | ↑^***^ | [M-H]- |
| 45 | Pisumionoside | C_19_H_32_O_9_ | HMDB0039947 | 403.2 | 5.66 | ↓^***^ | ↑^***^ | [M-H]- |
| 46 | 4,7-Dihydro-5-(4-methyl-3-pentenyl)-1,2,3-trithiepin | C_10_H_16_S_3_ | HMDB0038182 | 231.03 | 5.87 | ↓^***^ | ↑^***^ | [M-H]- |
| 47 | Enterolactone 3''-sulfate | C_18_H_18_O_7_S | HMDB0240506 | 377.07 | 5.64 | ↓^**^ | ↑^**^ | [M-H]- |
| 48 | Ipazine | C_10_H_18_ClN_5_ | HMDB0253563 | 244.13 | 5.81 | ↓^***^ | ↑^**^ | [M+H]+ |

**Table S3.** The reversed differential metabolites.

| NO. | Name | Formula | HMDB_ID | m/z | RT (min) | MOD vs. CON | HP vs. MOD | Adduct |
| --- | --- | --- | --- | --- | --- | --- | --- | --- |
| 1 | LysoPA(22:5(4Z,7Z,10Z,13Z,16Z)/0:0) | C25H41O7P | HMDB0114753 | 483.25 | 9.329 | ↑ | ↓ | [M-H]- |
| 2 | LysoPG(18:1(9Z)/0:0) | C24H47O9P | HMDB0240602 | 509.29 | 9.627 | ↑ | ↓ | [M-H]- |
| 3 | LysoPG(16:0/0:0) | C22H45O9P | HMDB0240601 | 483.27 | 9.348 | ↑ | ↓ | [M-H]- |
| 4 | LysoPG(18:2(9Z,12Z)/0:0) | C24H45O9P | HMDB0240600 | 507.27 | 8.955 | ↑ | ↓ | [M-H]- |
| 5 | PE(18:1(9Z)/0:0) | C23H46NO7P | - | 478.29 | 9.505 | ↑ | ↓ | [M-H]- |
| 6 | LysoPE(0:0/22:6(4Z,7Z,10Z,13Z,16Z,19Z)) | C27H44NO7P | HMDB0011496 | 526.29 | 8.825 | ↑ | ↓ | [M+H]+ |
| 7 | LysoPE(18:2w6/0:0) | C23H44NO7P | HMDB0011507 | 478.29 | 8.943 | ↑ | ↓ | [M+H]+ |
| 8 | LysoPE(0:0/16:0) | C21H44NO7P | HMDB0011473 | 454.29 | 9.282 | ↑ | ↓ | [M+H]+ |
| 9 | LysoPE(20:4(8Z,11Z,14Z,17Z)/0:0) | C25H44NO7P | HMDB0011518 | 502.29 | 8.88 | ↑ | ↓ | [M+H]+ |
| 10 | LysoPE(22:6(4Z,7Z,10Z,13Z,16Z,19Z)/0:0) | C27H44NO7P | HMDB0011526 | 524.28 | 8.831 | ↑ | ↓ | [M-H]- |
| 11 | LysoPE(22:4(7Z,10Z,13Z,16Z)/0:0) | C27H48NO7P | HMDB0011523 | 528.31 | 8.998 | ↑ | ↓ | [M-H]- |
| 12 | PC(20:3(5Z,8Z,11Z)/20:3(8Z,11Z,14Z)) | C48H84NO8P | HMDB0008377 | 834.60 | 10.96 | ↑ | ↓ | [M+H]+ |
| 13 | PC(18:2(9Z,12Z)/18:2(9Z,12Z)) | C44H80NO8P | HMDB0008138 | 804.55 | 11.218 | ↑ | ↓ | [M+Na]+ |
| 14 | PC(18:1(11Z)/20:5(5Z,8Z,11Z,14Z,17Z)) | C46H80NO8P | HMDB0008083 | 806.57 | 9.282 | ↑ | ↓ | [M+H]+ |
| 15 | PC(18:2(9Z,12Z)/20:4(5Z,8Z,11Z,14Z)) | C46H80NO8P | HMDB0008147 | 828.55 | 11.277 | ↑ | ↓ | [M+Na]+ |
| 16 | PC(14:1(9Z)/20:2(11Z,14Z)) | C42H78NO8P | HMDB0007913 | 756.55 | 11.182 | ↑ | ↓ | [M+H]+ |
| 17 | PC(20:4(8Z,11Z,14Z,17Z)/20:4(8Z,11Z,14Z,17Z)) | C48H80NO8P | HMDB0008477 | 852.55 | 10.949 | ↑ | ↓ | [M+Na]+ |
| 18 | PC(20:3(8Z,11Z,14Z)/16:0) | C44H82NO8P | HMDB0008397 | 784.58 | 11.404 | ↑ | ↓ | [M+H]+ |
| 19 | PC(22:6(4Z,7Z,10Z,13Z,16Z,19Z)/18:1(11Z)) | C48H82NO8P | HMDB0008728 | 854.57 | 10.542 | ↑ | ↓ | [M+Na]+ |
| 20 | PC(20:4(5Z,8Z,11Z,14Z)/18:1(9Z)) | C46H82NO8P | HMDB0008433 | 830.57 | 10.969 | ↑ | ↓ | [M+Na]+ |
| 21 | PC(20:3(5Z,8Z,11Z)/20:3(5Z,8Z,11Z)) | C48H84NO8P | HMDB0008376 | 856.58 | 10.998 | ↑ | ↓ | [M+Na]+ |
| 22 | PC(16:1(9Z)/16:1(9Z)) | C40H76NO8P | HMDB0008002 | 730.54 | 10.572 | ↑ | ↓ | [M+H]+ |
| 23 | PC(16:1(9Z)/18:2(9Z,12Z)) | C42H78NO8P | HMDB0008006 | 778.54 | 11.168 | ↑ | ↓ | [M+Na]+ |
| 24 | DG(18:2(9Z,11Z)/18:2(9Z,11Z)/0:0) | C39H68O5 | - | 639.50 | 11.259 | ↑ | ↓ | [M+Na]+ |
